# On the Treatment of the Diarrhœa of Typhoid Fever

**Published:** 1876-08

**Authors:** George Johnson

**Affiliations:** Senior Physician to King’s College Hospital, in Practitioner


### ON THE TREATMENT OF THE DIARRHŒA OF TYPHOID FEVER.

The diarrhœa of typhoid fever, as it is one of the most frequent symptoms of the disease, so is it one of the most troublesome, and one which often causes the greatest anxiety. It is a fact generally admitted that in the great majority of cases the severity and danger of typhoid fever are in direct proportion to the intensity and duration of the diarrhœa. Delirium and other serious cerebral symptoms, pulmonary engorgement, and renal congestion with albuminuria, are comparatively infrequent complications. The treatment of diarrhœa, then, forms a very important part of the management of the disease. During the many years of my connection with this hospital, I have had the opportunity of seeing the diarrhœa of typhoid fever treated in very different ways and with very different results, and I propose now to give you, in a few sentences, the results of my experience with reference to this important practical subject.

For a number of years the practice strongly advocated by Dr. Todd was generally adopted throughout the hospital. This consisted in persevering attempts to arrest the diarrhœa by repeated doses of opiates and other powerful astringents. It was then a common practice to give an enema containing from ten to fifteen or twenty drops of laudanum after each liquid stool. The result of this treatment, in a large proportion of cases, was that the diarrhœa continued in spite of the repressive treatment, and meanwhile the intestines were distended with gas, and the abdomen became tumid and tympanitic. Then the patients were tortured by the application of turpentine stupes to remove the tympanitis. The results were altogether unsatisfactory. Nor is it difficult to explain the failure of this opiate treatment. Without entering upon the consideration of disputed pathological theories, it can scarcely be doubted that one effect of opium must be to render the intestines torpid and to lessen their expulsive efforts, and as a result of this their putrid contents are retained until they decompose and give off noxious gases by which the bowel is distended and irritated, and so the diarrhœa perpetuated and increased. It is pretty certain that the healing of the ulcers must be impeded by the continual contact of the fetid morbid secretions, and that the distension of the bowel must cause pain and increase the risk of fatal perforation or rupture.

Now, for a number of years we have entirely changed our treatment, and I have gradually arrived at the conclusion that in the treatment of typhoid fever careful nursing and feeding are of primary importance, while, as a rule, no medicines of any kind are required, and when not required they are often worse than useless. The result of this change of treatment has been that diarrhœa is a less frequent symptom than formerly, and when it does occur it is far more tractable, while tympanitic distension of the abdomen is a rare event. The mischievous opiate enemata and the torturing turpentine stupes have disappeared together. I believe that one of the main reasons why we have less diarrhœa than formerly is, that we carefully abstain from the employment of irritating drugs of all kinds. As a rule, a fever patient has the "yellow mixture," which is simply colored water; and except an occasional dose of chloral to procure sleep, and a tonic during convalescence, we give no active medicines of any kind. We feed these patients mainly with milk, with the addition of beef-tea and two raw eggs in the twenty-four hours, and give wine or brandy in quantities varying according to the urgency of the symptoms of exhaustion, especially in the advanced stages of the disease; but in many of the milder cases, and especially in the case of children, we find that no alcoholic stimulants are required from the beginning to the end of the fever, and when not required they are of course best withheld. I have said that we give no irritating drugs of any kind. For a time I adopted the practice which has been strongly recommended, of giving repeated doses of diluted mineral acids. I have long since abandoned this practice, for I am sure that it was injurious, and it was injurious in a very obvious and intelligible way; it irritated the ulcerated mucous membrane of the intestines, it caused pain and griping, and I believe that it often increased the diarrhœa. I have no doubt that the comparative infrequency of severe and obstinate diarrhœa amongst my enteric fever patients during the last few years is partly attributable to the discontinuance of this mineral acid treatment. The extreme sensitiveness of the intestinal mucous membrane during the progress of typhoid fever is obvious and indisputable. It is admitted on all hands that the greatest care is required in returning to solid food during convalescence; a want of caution in this respect has often been followed by a return of pain and diarrhœa, an increase of temperature, and not seldom by a decided relapse. If, then, a slice of bread or a morsel of fish can excite such local and general disturbance even after the subsidence of the fever, how improbable is it that repeated doses of an irritating mineral acid can be given without injury during the height of the fever, when the ulceration of the intestines is actively progressing!

One more hint I wish to give you with regard to the diarrhœa of typhoid fever, which is, that in all probability it is often increased by the patient's inability to digest the beef-tea and eggs which are sometimes too abundantly given. When you have reason to suspect that this may be the case, I advise you for a few days to keep the patient entirely upon milk, which contains all the elements required for the nutrition of the tissues in a form most easy of digestion. I have had a large experience of the effects of an exclusively milk diet in various forms of disease. In many cases of Bright's disease it is very efficacious, but one of the inconveniences in some of these cases is its tendency to cause troublesome constipation. In many cases of chronic diarrhœa and dysentery, milk diet will effect a cure without the aid of medicines of any kind. There is now in Twining ward a girl, aged fourteen, who for four months had been suffering from dysenteric diarrhœa, the stools containing much blood and mucus. She was put upon a diet of milk alone, without medicine: within a fortnight the diarrhœa entirely ceased, and she is now convalescent. For the reason, then, that milk has this anti-laxative and even constipating effect in various morbid states, it is, when given alone, one of the best antidotes for the diarrhœa of typhoid fever.

That our treatment of fever cases is not unsuccessful is shown by the results. I find on reference to my case-books, that during the past year, from November 1, 1873, to October 31, 1874, I have had under my care in the hospital twenty-nine cases of fever; fifteen typhoid, and fourteen typhus. Some of the cases have been very severe, but all have been discharged well; not one death has occurred. This very satisfactory result I attribute mainly to the admirable nursing which our patients receive, and to our abstinence from mischievous medication. To only one of these patients was opium given, and that was for the relief of an irritable condition of bowel which remained after a very severe attack of typhoid. A few doses of opium soon put a stop to this, and the patient made a good recovery.—GEORGE JOHNSON, M.D., F.R.S., *Senior Physician to King's College Hospital, in Practitioner.*

